# Amyloid Fibrillation of Insulin: Amelioration Strategies and Implications for Translation

**DOI:** 10.1021/acsptsci.2c00174

**Published:** 2022-10-12

**Authors:** Megren H. A. Fagihi, Sourav Bhattacharjee

**Affiliations:** † School of Medicine, University College Dublin (UCD), Belfield, Dublin 4, Ireland; ‡ Clinical Laboratory Sciences Department, College of Applied Medical Sciences, Najran University, Najran 55461, Kingdom of Saudi Arabia; § School of Veterinary Medicine, University College Dublin (UCD), Belfield, Dublin 4, Ireland

**Keywords:** diabetes, insulin monomer, insulin hexamer, physical transformation, protein unfolding, protein agglomeration, amyloid degeneration, fibrillogenesis, hydrophobic interactions, translation

## Abstract

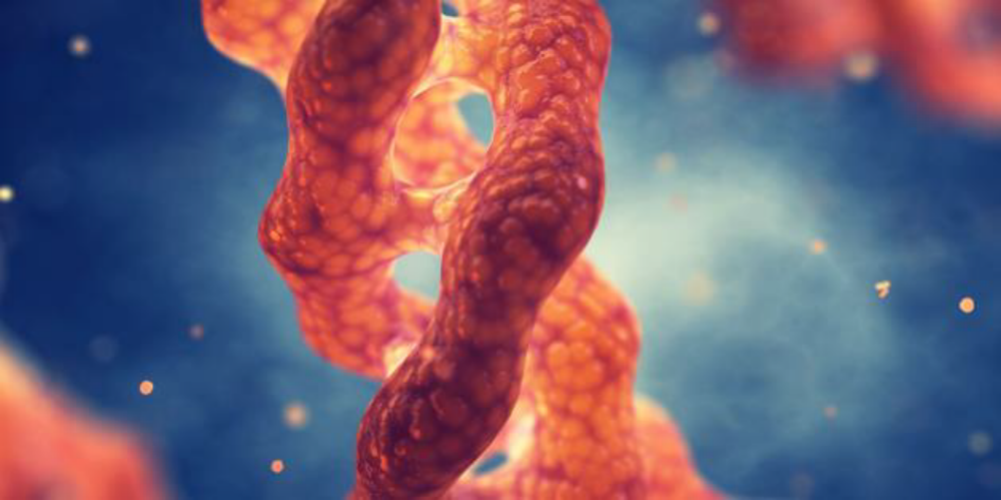

Insulin is a therapeutically relevant molecule with use in treating diabetes patients. Unfortunately, it undergoes a range of untoward and often unpredictable physical transformations due to alterations in its biochemical environment, including pH, ionic strength, temperature, agitation, and exposure to hydrophobic surfaces. The transformations are prevalent in its physiologically active monomeric form, while the zinc cation-coordinated hexamer, although physiologically inactive, is stable and less susceptible to fibrillation. The resultant molecular reconfiguration, including unfolding, misfolding, and hydrophobic interactions, often results in agglomeration, amyloid fibrillogenesis, and precipitation. As a result, a part of the dose is lost, causing a compromised therapeutic efficacy. Besides, the amyloid fibrils form insoluble deposits, trigger immunologic reactions, and harbor cytotoxic potential. The physical transformations also hold back a successful translation of non-parenteral insulin formulations, in addition to challenges related to encapsulation, chemical modification, purification, storage, and dosing. This review revisits the mechanisms and challenges that drive such physical transformations in insulin, with an emphasis on the observed amyloid fibrillation, and presents a critique of the current amelioration strategies before prioritizing some future research objectives.

Modern society is suffering from a surge of diabetes cases due to sedentary habits, consumption of unhealthy food with plenty of empty calories, underlying hypertension, obesity, smoking, and genetic predisposition.^1,2^ The silent pandemic of diabetes is now affecting all age groups while causing diseases such as diabetic retinopathy (Figure 1B), nephropathy, foot ulcers (Figure 1C), cardiovascular manifestations (e.g., stroke), and neuropathy.^3^ Emerging data have also linked diabetes with dementia.^4^ An estimated ∼10% of the global population is now affected by diabetes while under regular monitoring of capillary blood glucose (Figure 1D),^5^ while the mortality and morbidity due to diabetes have put the healthcare sector under stress.

**Figure 1:**
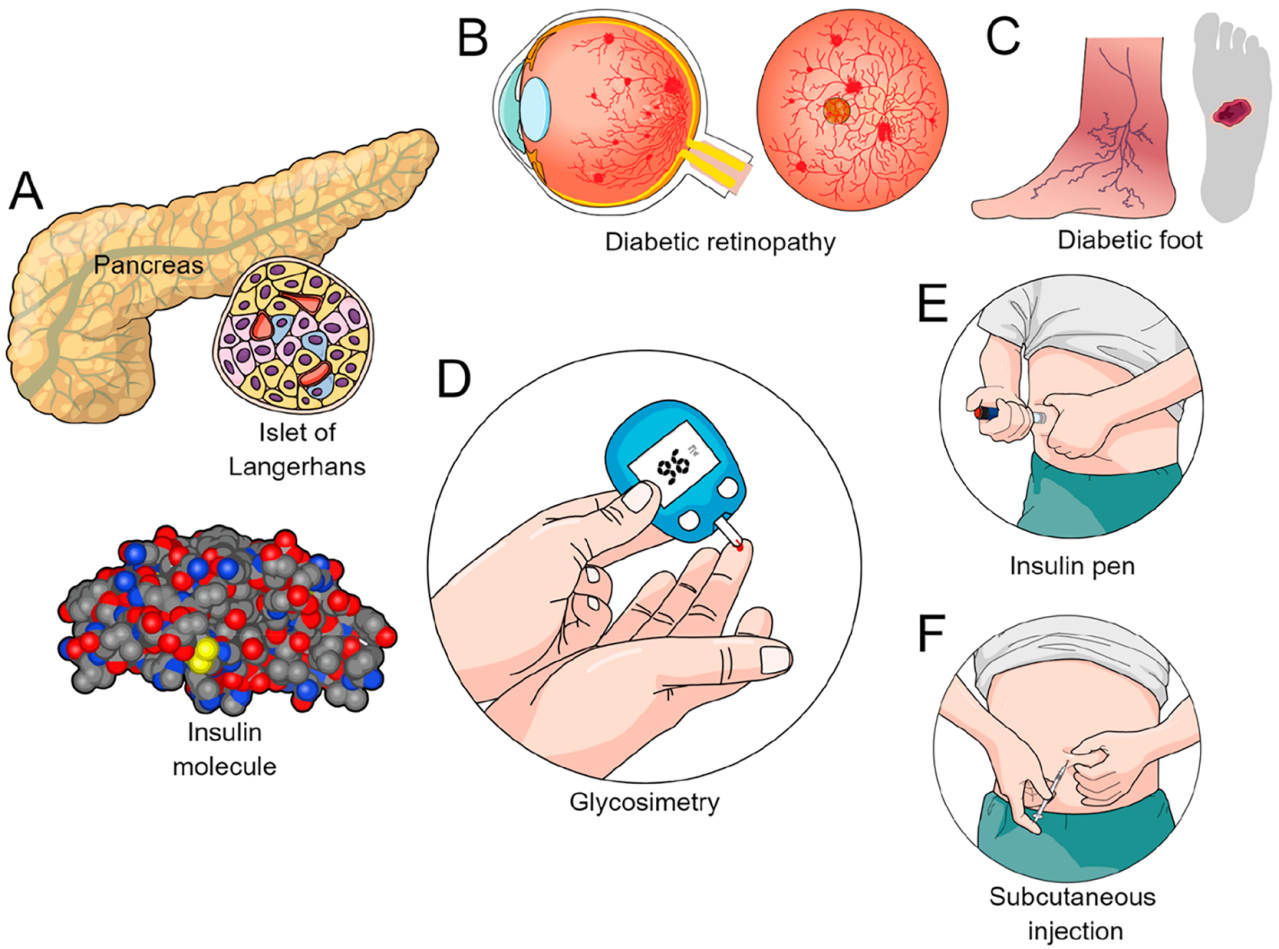
Overview of the insulin molecule, its delivery platforms, and complications associated with diabetes. (A) Insulin is released into the bloodstream from the β-cells of the pancreatic islets of Langerhans. (B) Lateral and anteroposterior ocular view in a case of diabetic retinopathy. (C) Foot ulcer found in diabetic patients. (D) Capillary blood glucose measurement (glycosimetry). (E, F) Insulin administration by (E) insulin pen and (F) subcutaneous injection.

Diabetes caused 4.2 million deaths worldwide in 2019^5^ and emerged as the seventh leading cause of death.^6^ Such a deteriorating landscape has naturally made insulin a therapeutically relevant biomacromolecule (Figure 1A). It is even more pertinent now as the global research community celebrates the centenary year of the discovery of insulin by the Canadian researchers Frederick Banting, Charles Best, John Macleod, and James Collip.^7,8^ It is also fair to recognize the seminal prior work by the Romanian physiologist Nicolae Paulescu that contributed to the discovery.^9^ Later, insulin became the first protein to be fully sequenced by Frederick Sanger (Nobel Prize in Chemistry, 1958). Unfortunately, the medical community is still searching for enteral formulations of insulin despite the advent of advanced delivery systems, including subcutaneous injections via pen (Figure 1E,F).

Injectable formulations of insulin, despite precise dosing, suffer from drawbacks like poor patient compliance and, important in the context of this review, the formation of amyloid fibrils^10^ that cause amyloidosis at the injection sites, noted commonly in Type II diabetes patients. Such amyloid fibrillation of insulin was reported as early as 1928,^11,12^ and is often noted as a subcutaneous lump with an immune response. The amyloid fibrillation also curtails the yield after purification *in vitro*.

Additionally, amyloid fibrils may result in poor dosing and hindrance to parenteral delivery. Although not reported in humans, insulin amyloidosis in the islets of the pancreas causing diabetes has been noted in the rodent degu (*Octodon degus*),^13^ found in Chile. Published reports have cautioned against the cytotoxic potential of amyloid insulin agglomerates due to oxidative stress caused by reactive oxygen species.^14,15^ Furthermore, an autoimmune response toward insulin fibrils has been linked with Parkinson’s disease.^16^

Such a tendency toward amyloid degeneration and formation of fibrils *in vitro* is also noticed when insulin is subjected to physicochemical alterations (e.g., fluctuations in pH, temperature, ionic strength), stirring/agitation, or exposure to hydrophobic surfaces (e.g., Teflon, polystyrene) due to a loss of secondary backbone.^17,18^ Insulin even elicits such degeneration after coming in contact with infusion apparatus, implantable pumps, and injectable pens.^19^ The insulin amyloid, although not an *in vivo* deposit in a strict sense, demonstrates a β-pleated structure, enhanced emission with a characteristic right shift with thioflavin-T (Th-T) fluorescence, and apple-green birefringence under polarized light upon staining with Congo Red dye.^20^

The physical transformation of insulin impacts its therapeutic strategy in the following ways: (i) it renders the dose, in full or partially, inactive or with a compromised physiological activity that is undesirable in diabetes patients; (ii) it is difficult, if not impossible, to model or predict such amyloid degeneration, which in turn aggravates the challenge of dose calculation; (iii) therapeutically it becomes difficult to access other routes of administration, including oral, nasal, or long-acting depot formulations, where encapsulated insulin formulations may address certain challenges, such as offering protection from an acidic (pH 2–3) gastric environment during oral delivery.

This discourse shall revisit the existing knowledge on such physical alterations of insulin with an emphasis on its molecular mechanisms, various factors that trigger such transformations, the ways to investigate the phenomena, and finally, the available strategies to mitigate them. The account will appreciate how such preventive measures can be incorporated within the existing therapeutic paradigms with anticipated challenges and prioritize strategies that can enhance the impact of existing insulin-based therapeutics with a fruitful translation.

## 1 The Insulin Molecule

**Figure 2:**
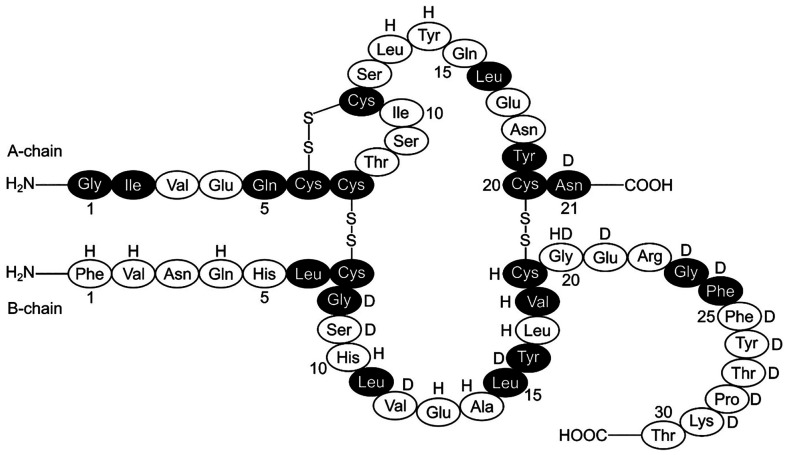
Human insulin molecule with its two polypeptide chains (A and B). The two interchain disulfide bonds (A7–B7, A20–B19) and one intrachain disulfide bond (A6–A11) are also shown. The darkened residues remain conserved across all the species. In porcine insulin, alanine replaces threonine (B30), whereas in bovine insulin, in addition to the substitution in porcine insulin, two additional substitutions are noted: alanine for threonine (A8) and valine for isoleucine (A10). The residues that help dimerization and hexamerization are marked with “D” and “H”, respectively.

Insulin (C_257_H_383_N_65_O_77_S_6_) is a globular protein and harbors a helical structure composed of A (21 amino acids) and B (30 amino acids) polypeptide chains (Figure 2).^21^ These chains are held together by two interchain disulfide linkages formed between the cysteine residues of A7–B7 and A20–B19.^22^ The molecular weight of an insulin molecule is 5808 Da.^23^ The A-chain has an intramolecular disulfide bond (A6–A11) and two antiparallel α-helices (A1–A8 and A12–A20), whereas the B-chain contains one such α-helix (B9–B19) flanked by dual turns and a flexible terminal (B21–B30).^24^

**Figure 3:**
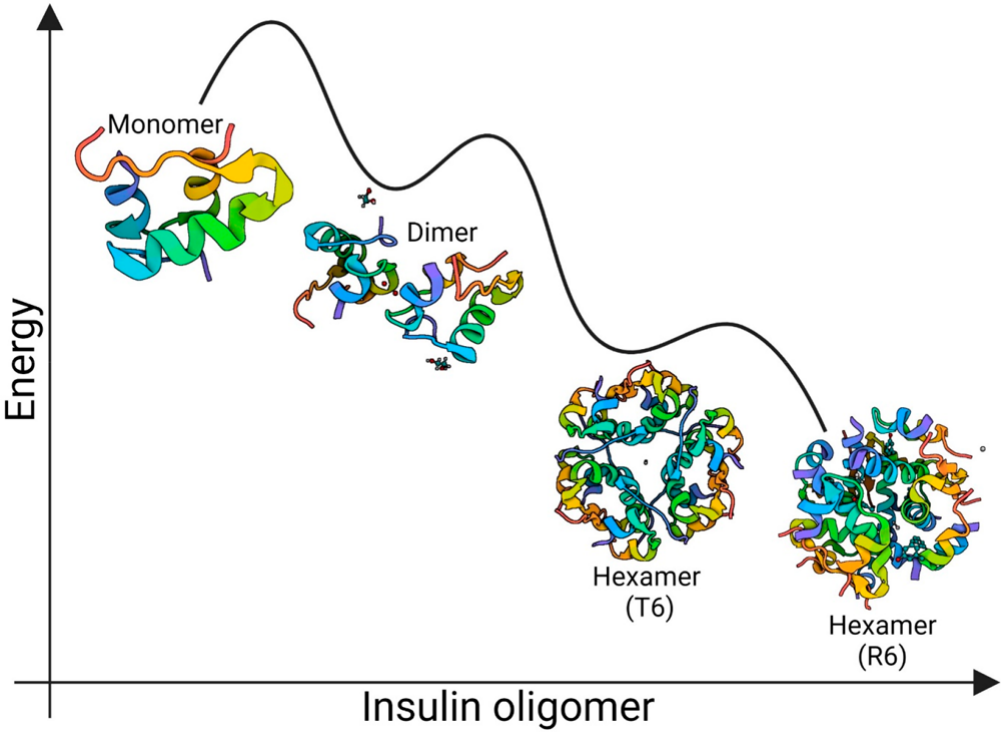
Thermoenergetic profile of insulin oligomers. Although physiologically active, the monomer (PDB: 3I40) is most unstable and quickly forms relatively stable dimers (PDB: 6S34). In the presence of Zn^2+^, the monomers gradually form the T6 hexamer (PDB: 1MSO), while adding both Zn^2+^ and *m*-cresol produces an even more stable R6 hexamer (PDB: 1EV6) bearing two extra chloride ions.

In humans, insulin is stored in the pancreas as an inactive and symmetric hexamer (∼36 000 Da) held together by two Zn^2+^ cations at the center of symmetry, surrounded by three molecules of water and six histidine residues (B10).^25^ Whereas the A-chain and helical segment of B9–B19 are stable, the regions of B1–B8 and B25–B30 are more flexible and vulnerable to manipulation. For example, adding phenol or its derivatives (e.g., *m*-cresol, resorcinol) in an insulin suspension introduces an extra helix at B1–B8 with ∼25 Å displacement of the phenylalanine (B1) residue (Figure 3).^26^ The hexameric insulin has two major conformational isomeric forms: the T6 isomer is produced by Zn^2+^ ions, and the R6 isomer is produced in the presence of both Zn^2+^ ions and phenolic compounds. The R6 isomer is thermoenergetically more stable than the T6 one (Figure 3).^27,28^

The *INS* gene of the pancreatic β-cells regulates the secretion of inactive pre-proinsulin (110 amino acids) that consists of a signal peptide connected to the A-, B-, and C-chains.^29^ Later, the signal peptide is cleaved in the rough endoplasmic reticulum by the signal peptidase enzyme, while the A- and B-chains remain connected by the C-chain, forming the proinsulin.^30,31^ As the proinsulin molecule folds, the A- and B-chains are connected by three disulfide linkages. However, the C-peptide is later cleaved by the endoprotease enzymes in the Golgi apparatus, and the cleaved C-peptide is then excreted in the urine.^32^ The remaining A- and B-chains—packed within the secretory vesicles in the Golgi apparatus—are subsequently processed by the proinsulin convertase and carboxypeptidase E enzymes that reduce the interchain disulfide bonds from three to two and activate the hormone as a monomer.^33^ The pancreas stores the molecule as a hexamer due to its enhanced stability.

The cavity inside an insulin hexamer (35 × 50 Å^2^) is spatially confined with a cross-section of 1.1 nm.^34^ The distance between the two Zn^2+^ cations is ∼1.4 nm, while the nanocavity is hydrophilic due to the glutamic acid (B13) and histidine (B30) residues, and holds 10 water molecules. Emerging data based on computer simulations, quantum calculations, and X-ray crystallography suggest that these confined water molecules contribute toward stabilization of the hexameric state by providing a dynamic interior while forming a robust network of hydrogen bonds with the nearby residues.^35^

A hydrophobic domain due to amino acids, such as phenylalanine, facilitates insulin dimerization, often by π–π interactions. It is worth noting that the free energy of formation for an insulin dimer and hexamer is −11.9 kcal mol^–1^ and −26 kcal mol^–1^, respectively.^36^ With an increase in blood sugar levels, the pancreatic β-cells release the hexamers into the blood, followed by their rapid dissociation into a dimer and, finally, the physiologically active monomer. Interestingly, while the inactive and torus-shaped hexameric form of insulin is rather stable, the active monomeric form is not. It only exists at a low concentration (≤0.6 μg/mL)^37^ and is fibrillogenic. The polymorphic amyloid fibrils bear a cross-β motif and are formed by the successive stages of oligomerization, nucleation, and growth.

## 2 Oligomerization of Insulin Molecules

Insulin molecules, when subjected to a variation of pH, temperature, or ionic strength, dealt with shaking/agitation, or mixed with organic co-solvents, are known to self-associate into oligomers as dimers, tetramers, or hexamers.^38^ Whether these partially unfolded oligomers contribute to fibrillogenesis remains an unsettled issue, although the current consensus is that they are hallmarks of the pre-fibrillar phase.^39^ Investigations based on X-ray crystallography, circular dichroism, and nuclear magnetic resonance have revealed that such (partially) unfolded monomers and dimers retain their native α-helix structure with an increase in both the random coils and flexibility of the chain termini. However, they lack any significant β-sheet structure due to a delayed α→β conversion.^40^

The isoelectric point of human insulin is 5.4,^41^ and the net charge of an insulin molecule varies depending on the pH. For example, the charges of an insulin monomer at pH values of <2 and 7.5 are +6 and −3, respectively.^42^ Insulin can thus be precipitated by setting the pH of its suspension within a range of 4.5–6.5. The role of acidic pH in triggering insulin fibrillation is important from a delivery perspective, for example, in infusion pumps, where the pH may fall due to a mixing of carbon dioxide and leached substances from the tubing.^43^ Moreover, it also creates challenges while storing insulin, where preservatives like methylparaben gradually hydrolyze into *p*-hydroxybenzoic acid.^44^

Other than pH, co-solvents are also known to influence insulin oligomerization. For example, in the presence of 20% (w/w) ethanol and acetic acid, insulin continues to sustain its monomeric form even at pH 2 and up to a concentration of 3 mg/mL (∼75 IU/mL).^45^ On the other hand, divalent cations, such as the Ca^2+^ and Zn^2+^, catalyze the formation of hexamers by imparting stability. Thus, in the presence of zinc, insulin hexamers start forming already at 0.6 mg/mL (∼15 IU/mL).^46^ On the contrary, without zinc, they form only at higher concentrations of ≥12 mg/mL (∼300 IU/mL).^37^

Fibrillar insulin comprises partially unfolded monomers, while a complete unfolding results in amorphous precipitates. Interestingly, unlike many other proteins, insulin is relatively thermoresistant and does not precipitate at temperatures as high as 100–140 °C.^47^ However, the effect of temperature on insulin fibrillation is often unpredictable and a culmination of a gamut of factors. For example, shear stress induces rapid fibrillogenesis in neutral insulin suspensions that are otherwise stable up to 60 °C.^48^

Crystallinity also plays a role in the thermosensitivity of insulin. While crystalline insulin in a suspension can tolerate higher temperatures, amorphous insulin is more vulnerable to temperature-induced fibrillation.^49^ Insulin also exhibits agglomeration when frozen, and upon thawing, the reconstituted suspension carries lumps and deamidated hydrolyzed products in acidic suspensions.^50^ In the presence of excess Zn^2+^, especially at a neutral or alkaline pH, insulin hexamers precipitate as crystals of various shapes, for example, cubic,^51^ tetrahedral,^52^ and rhombohedral.^53^ Both the monomeric and dimeric insulin have been crystallized.

## 3 Amyloid Degeneration of Insulin

The kinetics of insulin fibrillation has been investigated by various techniques, such as dynamic light scattering, small-angle X-ray scattering (SAXS), small-angle neutron scattering, atomic force microscopy (AFM), and Th-T fluorescence. The cumulative data have elicited that insulin fibrillation begins with a lag phase when no fibril is visible.^54^ The process gradually progresses toward a faster elongation/growth phase, followed by an equilibrium when the relative ratio between insulin monomers and fibril remains static.

**Figure 4:**
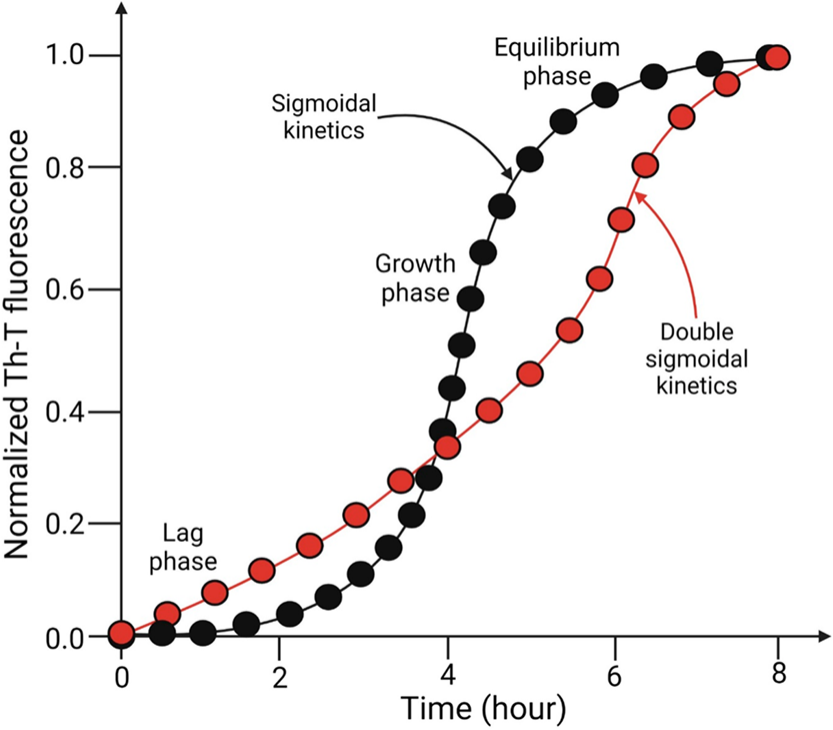
Sigmoidal (black) and double-sigmoidal (red) kinetics of insulin fibrillation passing through the lag, growth, and equilibrium phases—as determined by Th-T fluorescence.

Investigations based on Th-T fluorescence have revealed that, depending on physicochemical conditions, the sequence of lag, growth, and equilibrium phases demonstrates sigmoidal or double-sigmoidal kinetics (Figure 4).^55^ The agglomeration of unfolded insulin monomers is known to be energetically favorable. Hydrophobic interactions, van der Waals and electrostatic forces, hydrogen bonding, and solvation effects influence such agglomeration.^56^

## 4 A Mechanistic Overview of Insulin Fibrillogenesis

The mechanism of insulin fibril formation remains an unsettled issue, although the major consensus favors a nucleation-driven polymerization model.^57^ Such a mechanism proposes the formation of an unstable nucleus that provides a template for further addition of monomers, resulting in an extension of the fibrils.^58^ These primary nuclei are minuscule and comprise insulin dimers, trimers, or tetramers. The formation of such nuclei depends on a critical protein concentration^59^ and is able to skip the lag phase through less understood molecular interactions. However, adding insulin to the solution establishes a lag phase. A major drawback of such a nucleation-driven mechanism is its inability to explain the sigmoidal kinetics of fibrillation; instead, it depicts a parabolic curve.

Thus, rather than a primary nucleation-driven mechanism, the formation of secondary nuclei was proposed.^60^ It is a heterogeneous nucleation process where the fibrillation continues due to a range of processes, including fragmentation and branching, where nucleation to form new fibrils begins at the surfaces of already existing fibers. Time-lapse AFM studies have supported such a secondary nucleation model. It can explain the lag phase and sigmoidal nature of fibrillation in conjunction with the observed kinetics under pre-defined and confined reaction conditions.^61^

An intriguing observation is that insulin fibrillation becomes largely independent of concentration after a certain threshold (∼5 mg/mL).^62^ This finding indicates that the process follows a concentration-dependent nucleation mechanism where the formed nuclei keep gaining stability after a certain concentration. As a result, the existence of monomers in isolation becomes energetically untenable, and the fibrillation accelerates. Although such concentration-dependent nucleation has received endorsement from SAXS investigations, it still fails to explain the characteristic sigmoidal curve noticed during insulin fibrillation.

Perhaps there is no single mechanism that explains all the observations adequately. Some have even proposed an irreversible downhill polymerization^63^ that, instead of a nucleation-driven approach, follows a mechanism where the dissociation of insulin molecules into monomers is the rate-limiting step. Here, the monomers denote a higher energy state that pushes the equilibrium toward fibrils.

The role of insulin oligomers during insulin fibrillogenesis remains controversial. The oligomers vary widely in size: from a small globular geometry of 125 Å diameter to elongated forms of 200 Å to 1 μm.^64^ Some of them demonstrate a height commensurate to the insulin fibers, supporting the notion that such oligomeric species participate in fibrillogenesis. Investigations based on SAXS have demonstrated that, under an acidic condition with a mildly raised temperature, smaller oligomeric insulin facilitates the formation of mature insulin fibers in a concentration-dependent manner.^62^ Moreover, the helical shape of such oligomers renders them tailor-made for integration into the insulin fibers. The obtained data suggest the incorporation of such oligomers into insulin protofilaments^65^ with a probable role in fiber elongation. These smaller oligomers demonstrated a lack of native structure and a prevalence of β-sheets. The amyloid fibrils are insoluble in aqueous solvents and mineral acids. However, some reports indicate they dissolve and even renature under alkaline conditions (pH ≥ 11).^66^

## 5 Morphology of the Insulin Fibers

**Figure 5:**
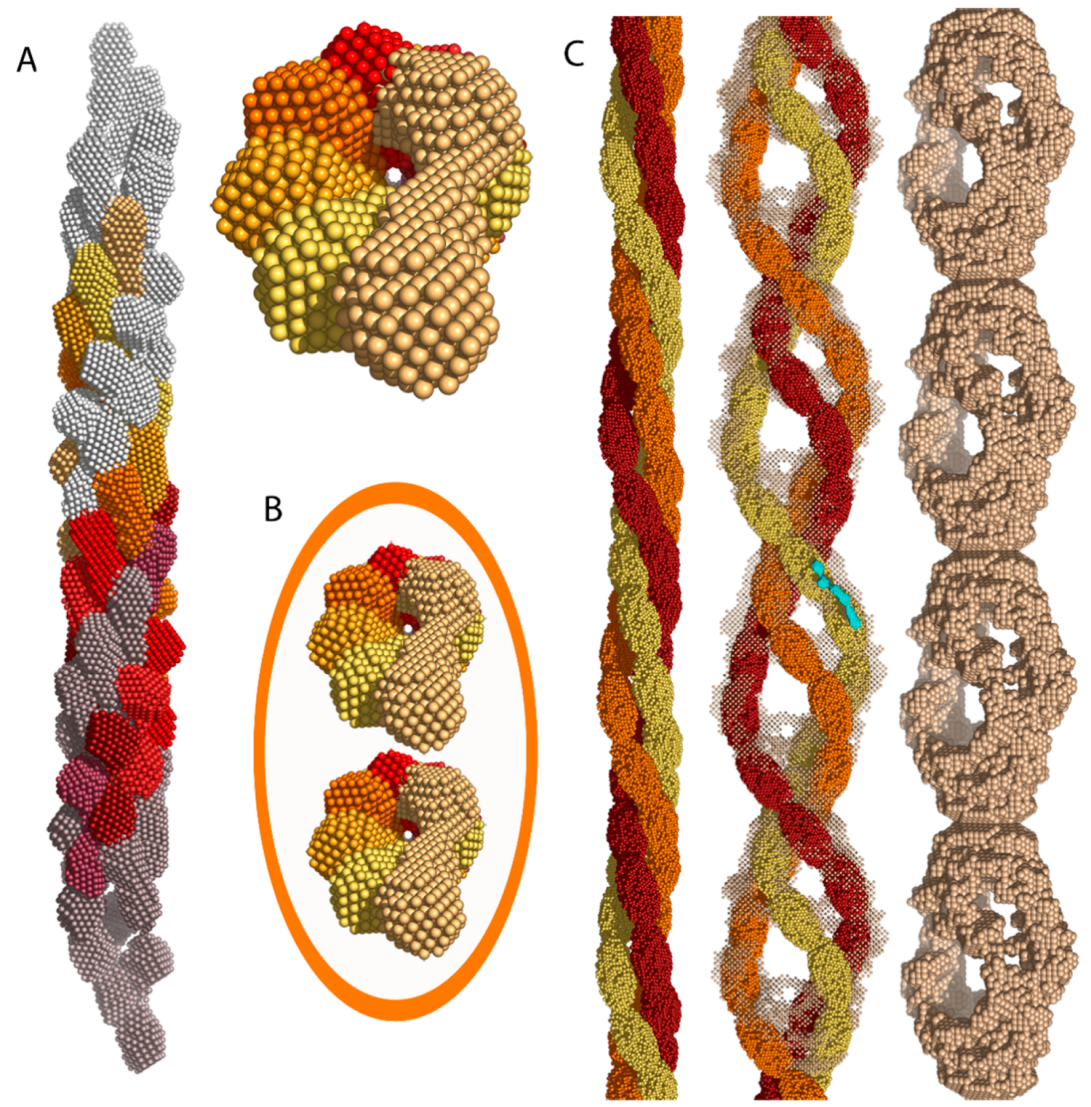
Successive stages of development in insulin oligomers: protofilaments, protofibrils, and fibrils. (A) Side and top views of an intertwined conglomeration of eight helical insulin oligomers (color scale: purple → red → light yellow) that form a protofilament. The gray segments at the open ends mark additional precursors. (B) Two intertwined protofilaments form a protofibril of 100 Å diameter. The orange ellipse shows the boundaries of an assembled protofibril. (C) Three protofibrils (orange, red, and yellow) interweave to form a mature insulin amyloid fibril. Both the side and frontal views are shown here. Reproduced from ref (62) under an open access Creative Commons License.

Insulin amyloid fibrils are known to demonstrate varied morphology and arrangements under the influence of fluctuating pH, ionic strength, and temperature. Investigations with X-ray crystallography, SAXS, AFM, and cryo-electron microscopy have provided valuable insights into the topic. A mature insulin amyloid fibril is often helical (left-handed),^67^ unbranched, and ∼100 Å in diameter,^68^ while its length may be up to a few μm (Figure 5).^69^ Such fibrils are composed of shorter units called *protofilaments* (length ∼40 Å, width ∼30 Å) composed of β-sheet polypeptide chains oriented perpendicularly to the fiber.^68^ It is thought that a cross-β spine is situated at the core of fibrils.^70^

Typically, 2–8 protofilaments form an individual fibril, and depending on the biochemical environment, it can be twisted or flat ribbon-shaped.^71^ The width of individual fiber bundles varies between 600 and 1000 Å, and the distance between crossovers in a fibril composed of two, four, and six helically twisted protofilaments is 525 Å, 355 Å, and 426 Å, respectively.^68^ Point mutations also impact insulin fibrillation. Hence, unlike the bovine insulin, where the amyloid fibrils demonstrate a helical arrangement, Asp(B10)-mutated human insulin under acidic conditions (0.1 M hydrochloric acid) forms laterally aggregated fibrils arranged in parallel bundles.^72^ Besides fibers, globular/spherical^73^ and circular agglomerates of insulin are also reported.^74^

The spherical insulin agglomerates are up to 50 μm in size and harbor a condensed core wrapped within a cloak of fibers.^73^ On the other hand, circular agglomerates develop under high-pressure conditions with diameters between 340 and 420 nm.^74^ The bovine insulin exhibits more fibrillation than the human and porcine ones due to the presence of hydrophobic alanine at the A8 position on the hexamer surface instead of the hydrophilic threonine in human and porcine insulin.

Due to a lack of imaging platforms that offer an atomic-level resolution, our knowledge of the molecular mechanisms of insulin fibrillogenesis remains nascent. Studies based on Fourier-transform infrared spectroscopy, circular dichroism, X-ray diffraction, and Raman spectroscopy have confirmed the gain in the β-sheet content in exchange for a loss in α-helices during insulin unfolding.^68^ Both the A- and B-chains contribute to fibrillation.

Cleaving the B-chain’s C-terminal accelerates fibrillogenesis. A repositioning of the hydrophobic moieties in an insulin monomer, viz., isoleucine at A2, leucine at B11, and leucine at B15—that are otherwise buried inside the monomer—by displacing the C-terminal of the B-chain might explain such a finding. Moreover, it also clarifies why insulin fibrillation is largely absent in proinsulin^75^ and mini-proinsulin,^76^ where the C-terminal of the B-chain is linked with the −NH_3_^+^ groups of the A-chain, thus restricting its mobility.

## 6 Preventive Measures

**Figure 6:**
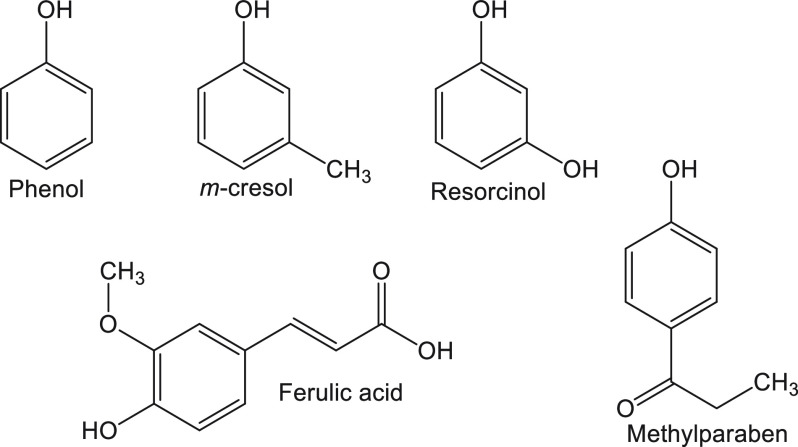
Chemical structures of phenol and phenolic compounds used as additives and preservatives in insulin formulations to impart stability.

The strategies for discouraging amyloid degeneration of insulin primarily rely on stabilizing the hexameric form and limiting the hydrophobic interactions. A common way to reinforce the hexameric form in a suspension of both short-acting (e.g., Actrapid, Humulin) and long-acting (Humalog) insulin is to mix zinc^77^ and phenolic compounds^78^ at a neutral pH (Figure 6). Conjugation of myristic acid to the lysine residue at the B29 position (e.g., Levemir) not only increases the plasma half-life of the formulation but also imparts stability by making the unfolded protein energetically unfavorable.^79^

Long-acting insulin formulations are less prone to fibrillation than short-acting ones. However, formulations like Lantus (glargine) are known to demonstrate fibrillation, although glargine was originally not formulated as a hexamer.^80^ Preservatives like methylparaben (Figure 6) are known to stabilize the crystallized forms, such as in ultralente insulin, ensuring a gradual release.^81^ Using a crystallized form of insulin is also a strategy for intermediate-acting isophane insulin. Dry powder formulations for nasal delivery, such as Exubera^82^ (Pfizer) and Afrezza (Sanofi)^83^—unfortunately now discontinued due to side effects and low sales—also demonstrated less fibrillation.

**Figure 7:**
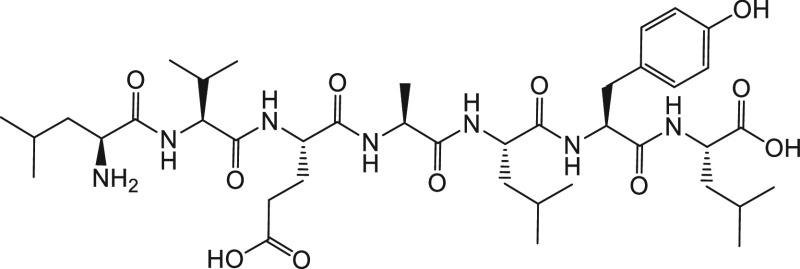
Chemical structure of the heptapeptide LVEALYL.

Bio-inspired peptides, such as the heptapeptide LVEALYL (Figure 7), which is part of the B-chain (B11–B17), have been used as anti-amyloidogenic agents.^84^ Interestingly, this heptapeptide sequence is known to self-fibrillate and even induce fibrillation at low concentrations. However, when present in excess, it retards fibrillogenesis. It is argued that at lower concentrations the heptapeptide favors the nucleation-driven insulin fibrillogenesis, whereas at higher concentrations it competes with insulin molecules in binding to the aggregated nuclei.

Other bio-inspired peptides with anti-amyloidogenic activity include RRRRRRLVEALYLV^85^ and NIVNVSLVK,^86^ with the latter noted to inhibit fibrillation in a dose-dependent manner. Synthetic peptides bearing tryptophan^87^ and taurine^88^ residues also deter insulin fibrillation. Short ferrocene-based peptide conjugates (e.g., ferrocene-Phe-Phe, ferrocene-Phe-Tyr) have also demonstrated their ability to limit insulin fibrillation and even dissolve the formed fibrils.^89^

Binding to hydrophobic surfaces, such as silane,^90^ inhibited insulin fibrillation at lower temperatures. Similarly, piperine-functionalized gold nanoparticles (∼10 nm) impeded insulin fibrillogenesis.^91^ The inhibitory effect of such hydrophobic surfaces toward insulin fibrillation might appear as a paradox, as such surfaces (e.g., Teflon,^92^ silicone oil,^93^ polystyrene^94^), on the contrary, are known to induce fibrillogenesis. It is argued that the hydrophobic surfaces blocking insulin fibrillation bind and subsequently mask the hydrophobic B23–B28 domain—a major driver of insulin agglomeration.

The chirality of the surface-grafted molecules also influences insulin fibrillation. For example, mica surfaces grafted with d-tartaric acid, when exposed to human insulin, elicited less fibrillation and reduced cytotoxicity *in vitro*.^95^ On the contrary, l-tartaric acid-grafted mica surfaces showed enhanced fibrillation and cytotoxicity. In a separate study, amyloid β-peptide, when incubated with insulin on a d-phenylalanine-functionalized mica surface, caused co-agglomeration of insulin with fibrous deposits and cytotoxicity against neuronal PC12 cells.^96^ On the contrary, l-phenylalanine-grafted surfaces under an identical experimental setup did not show fibrillation of comparable magnitude or cytotoxicity.

**Figure 8:**
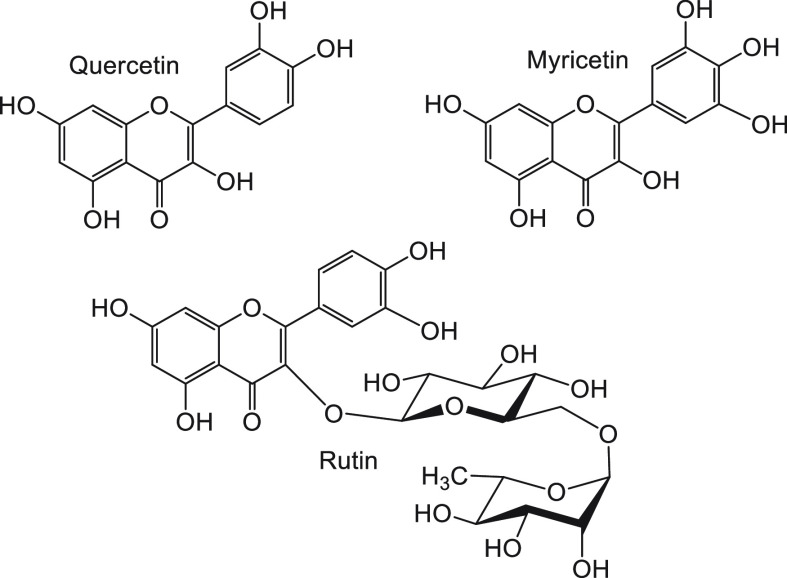
Chemical structures of the flavonoid compounds used to inhibit insulin fibrillation.

**Figure 9:**
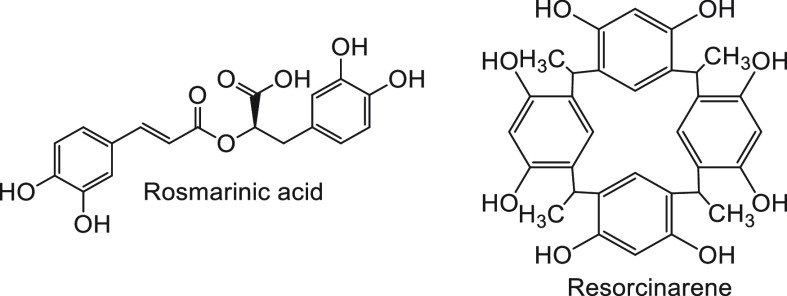
Chemical structures of polyphenolic compounds used to inhibit insulin fibrillation.

A gamut of natural compounds, such as flavonoids (e.g., quercetin,^97^ myricetin,^98^ and rutin^99^—a glycoside, Figure 8), polyphenols (e.g., rosmarinic acid^100^—inhibits insulin fibrillation by blocking dimer to monomer transition; resorcinarene^101^—a cyclic polyphenolic derivative of resorcinol, Figure 9), and phenolic compounds (ferulic acid,^102^ curcumin^103^), have been used to inhibit insulin fibrillation. Examples of other such natural compounds are ascorbic acid (vitamin C)^104^ and the anticancer drug paclitaxel.^105^ Additionally, gelatin has demonstrated an anti-fibrillogenic potential.^106^ These compounds not only can inhibit insulin fibrillation but also ameliorate the cytotoxicity of the fibers.

Besides, cyclodextrin,^107^ metal complexes (e.g., zirconium phthalocyanine, hafnium phthalocyanine, iron(II) clathrochelate),^108,109^ nanoparticles (e.g., carbon dots,^110^ fluorinated and magnetic core–shell nanoparticles^111^), and tetraphenylethene derivatives (e.g., 1,2-bis[4-(3-sulfonatopropoxyl)phenyl]-1,2-diphenylethene)^112^ inhibit insulin fibrillation through various mechanisms. For example, metal complexes intercalate within the early fiber grooves by stacking and inhibiting fiber elongation.

Hydroxyl- and acid-terminated carbon dots are known to engage the histidine residues at the B5 and B10 positions by electrostatic interactions, thus inhibiting fibrillogenesis.^113^ Similarly, magnetic γ-Fe_2_O_3_ nanoparticles (15.0 ± 2.1 nm) wrapped within a shell of fluorinated polymer poly(2,2,3,3,4,4,4-heptafluorobutyl acrylate), when incubated with insulin, demonstrated a full inhibition of fibrillation by stabilizing its helical backbone.^111^ Moreover, uncoated γ-Fe_2_O_3_ nanoparticles were shown to bind to insulin fibers which could be later used to separate the fibers by application of an external magnetic field.^114^ The thermostability of insulin has also been improved with the use of glycosylated insulin (e.g., disialo-glycoinsulin),^115^ the use of single-chain insulin (e.g., SCI-57^116^—a thermostable insulin analog where the A- and B-chains are connected by a glycine-rich peptide linker of six amino acids: GGGPRR), and the introduction of additional disulfide linkages between the A- and B-chains.^117^

## 7 Implications for Translation

The molecular instability of insulin toward even subtle changes in its biochemical environment entails significant challenges while preparing non-parenteral formulations, such as oral nanomedicines. Insulin suffers solubility issues and only dissolves in (mildly) acidic water. Unfortunately, an acidic environment favors fibrillation despite aiding solubility, especially after prolonged storage. Moreover, an acidic pH below insulin’s isoelectric point of 5.4 makes the protein cationic, which favors binding with a large range of biomacromolecules and surfaces that are anionic under physiological conditions.

Fibrillation is also evident when an insulin suspension is subjected to shaking, stirring, and heating. Hence, it is difficult to conduct wet lab procedures without disturbing the physical stability. As a result, an insulin mixture inadvertently bears some dissolved materials with insoluble fibers. Formation of insulin fibers represents not only a wastage of materials but also an undesirable reduction in yield. A fibril network in the reaction mixture hinders further reaction and causes difficulty in purification.

Moreover, it makes insulin delivery a challenge in resource-poor areas of the world, where sustaining a cold chain to preserve the molecular integrity of insulin formulations is difficult, if not impossible, at times. Unfortunately, a significant proportion of the global pool of diabetic patients now resides in tropical and developing areas of the world and are from impoverished backgrounds, with meager access to healthcare or refrigeration facilities to minimize temperature fluctuation or agitation that trigger insulin fibrillogenesis. Noticing the vacuum in the literature on how such lack of preservation impacts insulin delivery, there is enough space and reason to conduct studies investigating this issue, especially in remote areas and on deprived sections of humanity.

The physical transformations of insulin after coming in contact with hydrophobic surfaces make encapsulation difficult, especially from the perspective of oral delivery, where encapsulation within pH-sensitive materials, such as polymers, might be an option to safeguard the encapsulated payload from a highly acidic gastric juice followed by a release in the small intestine, the jejunum in particular, close to the Peyer’s patches.^118^ Regrettably, insulin tends to agglomerate after coming in contact with many of the polymeric candidates of encapsulation due to hydrophobic interactions that are difficult to predict, intervene, or contain.

Nanoformulations go through various maturation processes, such as Ostwald ripening,^119^ to gain thermodynamic stability—and the same is true for encapsulated insulin formulations. Hence, the particulate insulin formulations change in composition, with or without fibrillation and crystallinity over time. Unfortunately, such biochemical fluctuations are difficult to model, quantify, or compare, making the formulations untrustworthy from a delivery perspective.

Encapsulation in a typical core–shell particulate formulation condenses insulin molecules to form the cores, and the resulting spatiotemporal proximity between the molecules might trigger fibrillation.^120^ Over time, a part of this dense insulin core may get crystallized, which might provide stability. However, it is difficult to speculate how that will influence drug release. Furthermore, the coating layer, often composed of polymers, can catalyze fibrillation, especially at its interface with the core.

## 8 Future Perspectives

Knowledge about the physical transformations in insulin is almost as old as insulin’s discovery a century back. Surprisingly, despite such transformations being a widely studied and chronicled phenomenon, the research community is still struggling to find a solution to curb them. It is established now that the hydrophobic sites in insulin, such as the 10-residue-long hydrophobic patch at the C-terminal of the B-chain, facilitate fibrillation. It is prudent to note that a hexamer to monomer transition, which is necessary to exert a physiological effect, inevitably displaces this hydrophobic patch. Thus, it is a complex and, at times, paradoxical situation where the molecular reconfiguration that makes insulin physiologically useful simultaneously renders it vulnerable to deleterious physical transformations.

An interesting observation is that the physiologically active insulin monomer acts fast in the human body, leaving little time for fibrillation, agglomeration, or precipitation. It shows that any measure to slow down insulin fibrillation should shift the equilibrium toward hexamer or mask the hydrophobic patches. Unfortunately, none of these strategies is straightforward, well established, or free of the risk of curtailing insulin’s physiological impact or even making it defunct.

Adding excipients or organic molecules provides stability to insulin, although these external agents can compromise the biocompatibility of such formulations. Current insulin formulations frequently carry phenol or phenolic compounds that, apart from ensuring sterility, also promote the hexameric form in a suspension. However, these phenolic compounds are known to be toxic and cause neuronal symptoms by affecting the central nervous system. Exposure to hydrophobic surfaces inhibited fibrillation by engaging the hydrophobic pockets in insulin. Unfortunately, the balance between causing stability by masking the hydrophobic residues and stimulating fibrillogenesis by hydrophobic interactions is delicate, with little margin for error. Moreover, although such an approach might work *in vitro* under controlled lab conditions, a successful translation inside a human body remains elusive.

Given the molecular properties and behavior of insulin, it would be difficult to achieve stability and efficacy simultaneously. Engineering the insulin molecules, for example, by replacing some of the hydrophobic residues with hydrophilic ones might be an option. However, it risks compromising the physiological impact of the molecule. From an assay point of view, while discussing the bioactivity of insulin, the terms *active* and *inactive* are also not well defined and leave room for interpretation.

Fortunately, *in vitro* cellular assays are now available, for example, with insulin receptor-expressing hepatic HepG2^121−123^ and murine fat cells,^124,125^ to investigate the physiological activity of insulin. These assays provide an affordable, robust, and reproducible platform to investigate the bioactivity of insulin. They also provide numerical read-outs that can be used to quantify and compare different formulations from a functional perspective. Additionally, the inherent fluorescence of insulin molecules can be used to investigate its cellular uptake by microscopic tools. Such investigations can reveal how different insulin with various bioactivity behaves inside cells after uptake. The obtained data can be useful in recalibrating insulin activity and provide guidance while designing insulin therapeutics, where the challenge is to strike the right balance between stability and activity.

## 9 Conclusions

Despite earning the recognition of being the first peptide hormone to be discovered, fully sequenced, and synthesized by DNA recombinant technology, unfortunately, the parenteral route of administration continues to dominate insulin-based therapeutics, and endeavors toward developing enteral or nasal formulations have often resulted in frustrating outcomes. The molecular instability of insulin, especially its vulnerability to physical transformations due to alterations in heat, pH, ionic strength, additives, and hydrophobic surfaces, continues to hinder translation. The unchecked hydrophobic interactions facilitate the unfolding and, at times, misfolding of the polypeptide chains, resulting in agglomeration, fibrillogenesis, and precipitation. Such physical transformations result in curtailment of insulin efficacy with untoward therapeutic outcomes.

Current amelioration strategies include stabilization of insulin’s hexameric form by using additives, for example, zinc cations and phenol or its derivatives like *m*-cresol. However, such additives risk compromising the biocompatibility of the formulations. Other additives have also been tried, with a varied magnitude of success. Masking the hydrophobic pockets in the insulin molecule with synthetic peptides and mica surfaces functionalized with enantiomers has decreased fibril formation. However, such strategies need further investigation and a robust plan for translation from benchtop to bedside. Where applicable, relevant bioassays should be employed to assess the activity of insulin formulations. Future investigations should try to comprehend how insulin molecules behave in a biological milieu and gauge insulin’s physiological impact due to such transformations.
